# Gender equality in couples and self-rated health - A survey study evaluating measurements of gender equality and its impact on health

**DOI:** 10.1186/1475-9276-10-37

**Published:** 2011-08-26

**Authors:** Ann Sörlin, Lars Lindholm, Nawi Ng, Ann Öhman

**Affiliations:** 1 Dept of Public Health and Clinical Medicine, Epidemiology and Global Health, Centre for Global Health Research, Umeå University SE-901 87 Umeå, Sweden; 2 Umeå Centre for Gender Studies, Umeå University SE-901 87 Umeå, Sweden

**Keywords:** gender equality, health, index, gender differences

## Abstract

**Background:**

Men and women have different patterns of health. These differences between the sexes present a challenge to the field of public health. The question why women experience more health problems than men despite their longevity has been discussed extensively, with both social and biological theories being offered as plausible explanations. In this article, we focus on how gender equality in a partnership might be associated with the respondents' perceptions of health.

**Methods:**

This study was a cross-sectional survey with 1400 respondents. We measured gender equality using two different measures: 1) a *self-reported *gender equality index, and 2) a *self-perceived *gender equality question. The aim of comparison of the self-reported gender equality index with the self-perceived gender equality question was to reveal possible disagreements between the normative discourse on gender equality and daily practice in couple relationships. We then evaluated the association with health, measured as self-rated health (SRH). With SRH dichotomized into 'good' and 'poor', logistic regression was used to assess factors associated with the outcome. For the comparison between the self-reported gender equality index and self-perceived gender equality, kappa statistics were used.

**Results:**

Associations between gender equality and health found in this study vary with the type of gender equality measurement. Overall, we found little agreement between the self-reported gender equality index and self-perceived gender equality. Further, the patterns of agreement between self-perceived and self-reported gender equality were quite different for men and women: men perceived greater gender equality than they reported in the index, while women perceived less gender equality than they reported. The associations to health were depending on gender equality measurement used.

**Conclusions:**

Men and women perceive and report gender equality differently. This means that it is necessary not only to be conscious of the methods and measurements used to quantify men's and women's opinions of gender equality, but also to be aware of the implications for health outcomes.

## Background

### Gender and health

Men and women have different patterns of morbidity and mortality [1]. In Sweden, as in most other countries, women live longer (83.3 yrs in Sweden) than men (78.6 yrs in Sweden) but report more ill-health and have higher healthcare utilization [2]. These differences between the sexes present a challenge to the field of public health. The question why women experience more health problems than men despite their longevity has been discussed extensively [3-5], with both social and biological theories being offered as plausible explanations. In this article, we focus on how gender equality in a partnership might be associated with respondents' perceptions of health.

All societies today are characterized by a gender system; sex is one important (sometimes the most important) dimension when privileges and burdens are divided. Data from all over the world show that women have fewer socioeconomic privileges than men, and this uneven distribution of course influences health.

The status syndrome theory claims that health disparities can to a large extent be predicted by inequality, hierarchies and social isolation [6]. According to this theory, health depends on the social environment, and particularly on our sense of autonomy and control. Marmot argues that gender inequities damage the health of millions of girls and women through unfair divisions of work and leisure, differences in decision-making power and overall differences in possibilities of improving one's life [7]. We agree that gender inequalities negatively impact women's health; however, we wish to add that men are losers, too. Masculinity is often demonstrated by risky behaviours such as heavy drinking and careless driving [8]. The mortality differences between men and women are greatest in young adults, mainly explained by an excess mortality among males through causes such as injury and violence, often connected to alcohol [9]. However, there is a trend - at least in Western societies - for men to adopt healthy lifestyles similar to women's, such as consciousness about the body and its functions, and for women to adopt less healthy behaviour, such as smoking and increased alcohol consumption. These behavioural changes seem to result in a convergence in health patterns. Sex differences in longevity in Sweden were about six years in the 1970s but have now decreased to about four years [9]. Lung cancer has increased among women but decreased among men, and utilization of hospital care is now at the same level for men and women in the 45 to 64 years age group [9]. Although the change in the mortality pattern is obviously connected to changes in smoking and drinking patterns, it could be argued that steps towards increased gender equality are an underlying cause. Being a present and committed father may reduce mortality risks [10], as a caregiver is more risk adverse than a breadwinner.

A more detailed prediction of these changes towards long-term convergence in health patterns is very difficult because the development of gender relations is not symmetrical. Women expanded into the public sphere, taking on paid work outside the household, before men expanded into the household sphere. At this stage, women bear a double work burden, which might produce radically different health consequences compared to a more equal state in which the presence of men and women is a given in both the public and domestic spheres.

The eco-social theory advances embodiment as the central construct, recognizing that humans are simultaneously social beings and biological organisms [11]. Outlining the eco-social theory of disease distribution, Krieger situates both population health and epidemiological theory in a societal and ecological context. She includes not only the biological differences between men and women but also the possible impact on gendered health patterns of the differently defined social groups to which men and women belong. Krieger writes: "If any on-average difference between women and men is observed, it might arise from gender relations, not just sex-linked biology, or perhaps both, synergistically" [12]. The macro and micro levels of people's health are tied together by the practical outcomes of both "Doing gender" and "Doing health" - at work, in their relationship and in health care systems. This practical outcome then manifests itself in our bodies i.e. in a variety of embodied ill-health. Human bodies are an inextricable mix of biological conditions and acquired accustomedness [13].

### Gender equality in couples

Most variables measuring social position are strongly correlated ("high education", "high income", "social prestige" etc.). This consistency also appears in gender analyses (women works more part time, have lower incomes and less social prestige etc.). However, an analysis of gender equality in a partner relationship breaks this pattern: the unit under study is a couple often characterized by both longer and shorter education, high and low income etc. These uneven distributions of both resources and burdens between the two partners constitute the inequitable relationship.

According to Krieger, the disadvantaged party in a non-gender-equal couple relationship (most often the woman) will embody this gendered inequality, emanating from weaker economic resources, less power to decide on private matters, lower prestige and so on (pp. 214). Krieger argues that this embodied lack of power will manifest itself as ill health.

Determinants of current and changing societal patterns of disease distribution including health inequities are: (1) exogenous to people's bodies and (2) manifest at different levels, involving different levels of society from macro level to micro level [12]. Krieger also mentions the health care system as a potential actor in the creation of health inequities. Risberg et al. have demonstrated this gender bias in health systems, for example as unequal treatment of women and men and gender-biased counselling [14]. Differences in health status between population groups can causally result from group relations rather than intrinsic biology, even though the biological differences are manifested in individual bodies (Krieger pp. 215). Extending the idea of embodiment to include not only the societal interaction but also the biological processes makes it possible to contextualize the comparison between men and women, i.e. comparing groups rather than individuals. This comparison between men and women will reveal two groups with different conditions for health.

In order to establish a normative definition of "equality" in a couple, we decided to take official Swedish National Gender Policy. The choice of Swedish policy as the starting point of the project means that we accept the following definition of "gender equality": the situation where men and women have the same rights and possibilities to shape society and their own lives, regardless of their sex. In such a society, gender in many situations would be irrelevant. This has been described by Moller-Okin as the genderless society: "*A just future would be one without gender. In its social structures and practices, one's sex would have no more relevance than one's eye colour or the length of one's toes*" [15]. In such a society, men and women would participate in more or less equal numbers in every sphere of life, from infant care to high-level politics.

At the core of politics and academic disciplines such as philosophy, economics, public health and even medicine, the question of the allocation of desirable goods (power, income, health etc.) and burdens (taxes and possible national defending responsibilities etc.) is central. Assessing the fairness of a certain distribution requires some measurement of how goods and burdens are allocated between individuals. For this purpose, two main methods have evolved: either the individuals assess their position themselves (in happiness or quality or life or health) or an external observer performs the assessment (belonging to the poorest quintile etc.). Since both methods have their merits, we decided to use self-perceived assessment and to complement it with a new model. Although the new model, here called self-reported gender equality, is not an external assessment, it is nevertheless different from the single question of self-perceived gender equality, as it measures how the couple practice gender equality in their everyday life and household duties. A comparison of these two measures is conducted in the current study. "Self-perceived assessment" was simply a question asking: "How do you rate the gender equality in your relationship?" For the self-reported assessment respondents were asked to report, for themselves and their partners, on three domains: 1) background variables, 2) sharing of time and responsibilities, and 3) sharing of parental leave. These three domains were then used to generate an index. Self-perceived assessment of gender equality is a simple measurement, though not always a trustworthy one, as "gender equality" has an imperative tone in Swedish society, making an overrating bias likely. Self-reported assessment of equality is not an easy or trivial task either, as all indices have to deal with the choice of domains and aggregation rules. However, comparing measurements extends the possibilities of a broad understanding of phenomena.

### Measuring health

We use Self-Rated Health (SRH) as the health outcome in this study. SRH is an important and frequently used health outcome in health surveys [16]. One of the most common methods for SRH is a single question asking people to rate their overall health on a scale from excellent to poor. It is argued that this simple global question provides a good summary of how people perceive their overall health [16,17]. This global self-rated health assessment is valuable because it is sensitive to health changes, captures broader dimensions of health than traditional diagnostic tools and is easy to manage. The self-rated health indicator has been found to have good reliability [18-21] and has also been recommended by the World Health Organization [22].

### Aim

The main aim of this study was to analyse the association between gender equality in a partner relationship and self-rated health. The study also evaluates the impact of the gender equality measurements used.

## Methods

### Study participants

This study was a cross-sectional survey, based on a population drawn from a previous register study. The register study comprised 1.1 million people working in 8000 companies in Sweden. The companies were ranked according to the Organizational Gender Gap Index (OGGI) [23]. Based on Swedish gender policy and information available in public registers, six variables were chosen to form the OGGI. The purpose of the register-based index was to provide a practical tool for gathering information on gender equality at organizational level. The most gender-equal and the least gender-equal companies, in total 21, where invited to take part in the present study. All registered employees of the 21 companies, 1885 men and 976 women, were invited to participate by completing a self-administrated questionnaire. A total of 1407 individuals responded to the survey, 62.2% men (875) and 37.8% women (532), yielding a response rate of 49% (46.4% for men and 54.5% for women). After excluding respondents who had subsequently retired (n = 43), respondents without a partner (n = 295), and respondents who did not answer all the variables needed for further analysis (n = 397), a total of 685 individuals with complete information were included in the analysis, 439 men and 246 women.

There are some differences between our study population and the general Swedish population, for example in education. Swedish women have higher average educational levels than men, but in this study this is not the case [24]. This could be due to a relatively high mean age of participants in our study. The reason why we have more male participants in the study is that the private sector employs more men, meaning that more men were employed in the participating companies. As in most Swedish studies of this kind, the response rate is higher for women, participants with a high education level, older participants and people on high incomes.

### Instrument and variables

The survey questionnaire was constructed in collaboration with Statistics Sweden, who later administered the data collection and entry. The questionnaire was first tested for user-friendliness in a pilot study with 300 people and subsequently revised with the help of experienced constructers at Statistics Sweden. We measured gender equality using two different measures: 1) a *self-reported *gender equality index, and 2) a single *self-perceived *gender equality question. In this way, we aimed to analyse the consistency in the reporting of gender equality by contrasting the two measurements.

The construction of the self-reported gender equality index follows a long tradition in the gender equality assessment field. Indices often contain the same kinds of variables, such as education level, income, employment status and health outcomes [25-27]. In this index, we aimed to measure the gap between the two people in the relationship.

First, the questionnaire gathered information on *self-reported gender equality *for three domains, for both the respondent and his/her partner. These domains were (1) education, income, and full or part-time employment; (2) sharing of time and responsibilities for household work; and (3) sharing of parental leave following the birth of a child, and sharing of temporary parental leave for child sickness. This set of variables was chosen firstly as the variables resemble those used in other studies of gender equality [25,28-32], and secondly because they are almost the same as those used in our previous register study. In the survey study, we additionally asked about sharing for household duties and responsibilities. Each respondent completed a form, in which they were asked to answer the questions and to provide information on both themselves and their partner. We measured the gaps in the variables between respondents and their partners, and used the results to generate a self-reported gender equality index.

Educational achievement was measured by asking respondents about their and their partner's highest level of education, using three categories: compulsory education (secondary education), high school (further education), and university/college (higher education). Respondents were also asked about their and their partner's net income, with responses recorded in five income categories. Employment was measured by asking whether respondents and their partner worked full time (90-100%) or part time (less than 90%). We then measured similarities and differences in education, income, and full or part-time employment between respondents and partners. Three new dichotomous variables were constructed, describing whether the respondent and his or her partner had equal or differing responses for each variable. We also asked how respondents and their partner shared unpaid household work. This included cleaning, cooking, washing dishes, routine household shopping, laundry, maintenance of the home, looking after the car, dropping off and picking up children at or from school or daycare, routine meetings at school and health check-ups, children's leisure activities, caring for elderly relatives, and planning of household duties. The response categories were as follows: the respondent does most of the household work, their partner does most of the household work, they share the household work equally, or household work is not relevant in their setting. In the analysis, after excluding those for whom household work was not relevant in their setting, we calculated the proportion of total household work that was shared equally. Respondents who shared at least 50% of the variables measuring household work were considered gender equal.

In two separate questions we asked who **- **the respondent or his/her partner - took longer parental leave and temporary parental leave. Their responses were re-coded into three categories: the respondent stayed at home more often, his/her partner stayed at home more often, or they shared parental and temporary parental leave equally. We then constructed two new dichotomous variables describing whether each type of leave was shared equally or not. Finally, we combined all these six new dichotomous variables to generate an index representing gender equality. As with other studies measuring gender gaps, including the preceding register study, no consideration was taken of its direction [33].

Secondly, we measured *self-perceived gender equality *by asking respondents to indicate their perceived gender equality with their partner in the relationship. Self-perceived gender equality was assessed on a modified four-point categorical scale by asking respondents the following question: "How do you rate the gender equality in your relationship?" The response alternatives were "completely gender equal", "relatively gender equal", "not very gender equal" and "not at all gender equal". We categorized the responses into three groups: completely equal (i.e. those who answered "completely gender equal"), relatively equal (those who answered "relatively gender equal"), and not equal (the other two categories on the categorical scale).

The outcome measure in this study was self-rated health, where subjects assessed their health as excellent, good, fair or poor. "Fair" and "poor" health ratings were combined into a single group, referred to henceforth as poor-rated health; the "excellent" and "good" categories were also combined and represent the reference category.

### Statistical approach

We generated a self-reported gender equality index using principal component analysis (PCA). PCA has been used extensively to develop a wealth index as a proxy for socioeconomic status [34,35]. In our study, PCA was used to reduce the dimensions of the six intercorrelated self-reported variables into one or more uncorrelated components. We tested the basic assumption of PCA, i.e. independent sampling and linear correlation between the variables used. The Kaiser-Meyer-Olkin measure of sampling adequacy was 0.6, indicating that our sample size was adequate for the PCA. The determinant of the correlation matrix of 0.79 and Bartlett's test (chi-square = 162, df = 15, p < 0.001) indicated that all six variables included in the analysis were correlated with each other. In the PCA, we selected the first two components, which captured and explained 46% of the variation in the data. We calculated the factor scores from these components and categorized the scores into tertiles, with the lowest tertile representing couples with the least gender equality and the highest tertile representing those with the greatest gender equality. This index is subsequently termed the "self-reported gender equality index".

We assessed the agreement between self-perceived and self-reported gender equality using the weighted kappa statistic and its 95% confidence interval (CI). The weighted kappa statistic was used to accommodate the ordinal nature of the response scale, and quadratic weight was used in the calculation [36]. We conducted simple logistic regression to get a crude estimate of odds ratio for gender equality in relation to self-rated health. We continued with multivariable logistic regression to assess the association between gender equality and self-rated health adjusted for respondent's age, education, occupational grade and income. We constructed separate models for self-perceived gender equality and the self-reported gender equality index. For each model, we reported the odds ratio and its 95% confidence interval (95% CI). All the data analyses were conducted in STATA Version 11 (StataCorp 2009).

### Ethical considerations

Ethical approval was obtained from the Regional Ethics Review Board in Umeå (D-no. 06-156 M).

## Results

A total of 685 respondents were included, 439 (64%) men and 246 (36%) women. Men in this study had slightly higher educational levels than women, differing from the general pattern in Sweden where women have recorded higher education levels since 2000. This different pattern could be due to the chosen sectors, the mean age of the respondents, or both. The distribution between men and women in the variables income and employment correspond more to the usual pattern. The group of men aged 50 years or older is slightly larger than the group of women in the same age group, which could be part of the explanation for the variables discussed above. Background variables for both respondents and their partners are shown in table 1.

**Table 1:** Distribution of study subjects and their partners

Variables	Respondents		Partners	
	
	Men (n = 439)	Women (n = 246)	Men (n = 247)	Women (n = 438)
Age Group (%)				
< 30	22 (5.0)	14 (5.7)	9 (3.7)*	38 (8.7)
31-50	315 (71.8)	183 (74.4)	171 (69.5)	319 (73.0)
> 50	102 (23.2)	49 (19.9)	66 (26.8)	80 (18.3)
Education (%)				
Secondary	49 (11.2)	37 (15.0)	40 (16.2)*	26 (5.9)
Further	164 (37.4)	96 (39.0)	98 (39.7)	165 (37.7)
Higher	226 (51.5)	113 (45.9)	109 (44.1)	247 (56.4)
Income (%)				
< 20,000	23 (5.2)*	64 (26.0)	29 (11.7)*	143 (32.7)
20,000 - 30,000	135 (30.8)	87 (35.4)	107 (43.3)	197 (45.0)
> = 30,000	281 (64.0)	95 (38.6)	111 (44.9)	98 (22.4)
Employment (%)				
Full time	421 (95.9)*	176 (71.5)	234 (94.7)*	267 (61.0)
Part time	18 (4.1)	70 (28.5)	13 (5.3)	171 (39.0)

* indicates a statistically significant (p < 0.001) difference in the distribution of the background variable in men and women using the chi-square test

The self-reported gender equality index was derived from three domains. The first domain included equality in education, income and fulltime/part time employment. The second domain, comprising 11 questions, dealt with equality in the proportion of time and responsibility for household work. The last domain examined equality in the sharing of parental leave and temporary parental leave. The results showed that two-thirds of men and women reported equality in educational level. Men worked full time more often than their partners and they also earned more. Women reported significantly more gender equality between themselves and their partner for income and education (p ≤ 0.05).

Table 2 presents the six variables constituting the self-reported index. Overall, only about 17% of respondents reported sharing household work equally. About 43% of the respondents reported equal sharing of temporary leave for child sickness; only about 13% of respondents reported sharing parental leave equally.

**Table 2:** Gender equality between respondents and their partners as reported by male and female respondents

Variables	As reported by respondents	
	
	Men (n = 439)	Women (n = 246)
Equality of education level (%)		
Equal	296 (67.4)	167 (67.9)
Unequal	143 (32.6)	79 (32.1)
Equality of income (%)		
Equal	127 (28.9)*	127 (51.6)
Unequal	312 (71.1)	119 (48.4)
Equality in full-time employment (%)		
Equal	272 (62.0)*	181 (73.6)
Unequal	167 (38.0)	65 (26.4)
Proportion of household work shared equally (%)		
Equal	73 (16.6)	44 (17.9)
Unequal	366 (83.4)	202 (82.1)
Parental leave (%)		
Equal	59 (13.4)	32 (13.0)
Unequal	380 (86.6)	214 (87.0)
Temporary parental leave (%)		
Equal	189 (43.0)	106 (43.1)
Unequal	250 (57.0)	140 (56.9)

* indicates a statistically significant (p < 0.001) difference in the distribution of the variables in men and women using the chi-square test

Men and women reported differently on how they shared their parental leave and temporary parental leave. Only 1.8% of men reported being the one that stayed at home most during parental leave, in contrast to the 86.6% of women who reported doing so. The same patterns, though with smaller differences, were observed when respondents were asked about temporary leave: only 8.4% of the men reported staying at home most in contrast to about 52% of the women who reported doing so.

### Self-perceived gender equality

There was a clear difference between how men and women perceived and reported gender equality. Approximately 43% of the men and 28% of the women in this study perceived their relationship with their partner to be completely gender equal; only 4% of the men and 13% of the women respectively reported their relationship as not equal. However, despite the fact that more than 80% of the women considered their relationship to be completely or relatively gender equal, more than 80% took the greater part of parental leave.

**Figure 1:**
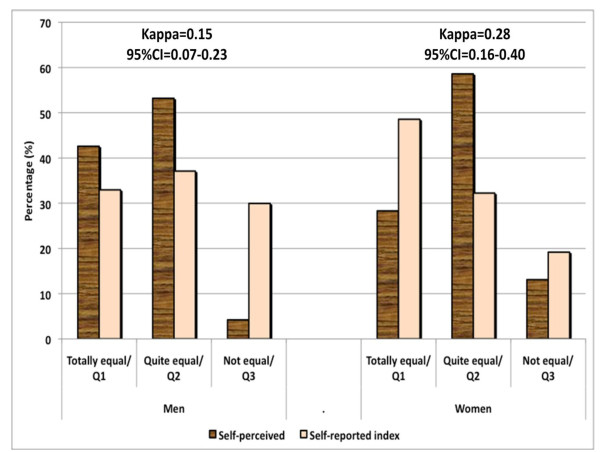
**Agreement between self-perceived gender equality and self-reported gender equality index**.

Overall, we found little agreement between the self-reported gender equality index and the self-perceived gender equality measure. These results were consistent for both men and women (with kappa statistics between 0.15 for men and 0.28 for women), even though the agreement was slightly better and different for women than for men (Figure 1). The patterns of agreement between self-perceived and self-reported gender equality were quite different for men and women. Men perceived more gender equality than they reported; in contrast, women perceived less gender equality than they reported in the index. Only 30% of women perceived their relationship with their partner to be completely equal, while almost 50% of the men belonged to the most equal quintile.

### Gender equality and self-rated health (SRH)

Overall, 82% of the men and 78% of the women reported their health as good (table 3). The upper half of the table shows the relation between SRH and the self-reported equality index, and the lower half the relationship between SRH and self-perceived equality.

**Table 3:** Association between gender equality and self-rated health

Variables	Self-Rated Health			
	
	Men		Women	
	
	Good health	Poor health	Good health	Poor health
**Overall self-rated health, n(%)**	359 (82)	79 (18)	190 (77.9)	54 (22.1)

**Self-reported equality index, n(%)**				
1^st ^tertile (most equal)	120 (84.5)	22 (15.5)	97 (82.2)	21 (17.8)
2^nd ^tertile	130 (80.8)	31 (19.3)	59 (74.7)	20 (25.3)
3^rd ^tertile (least equal)	105 (80.8)	25 (19.2)	33 (71.7)	13 (28.3)
**Unadjusted OR (95% CI)**				
1^st ^tertile (most equal)	1.30 (0.69-2.44)		1.82 (0.82-4.04)	
2^nd ^tertile	1.00 (0.56-1.79)		1.16 (0.51-2.63)	
3^rd ^tertile (least equal)	1		1	
Adjusted OR (95% CI)*				
1^st ^tertile (most equal)	1.03 (0.53-2.00)		1.54 (0.6-3.95)	
2^nd ^tertile	0.88 (0.48-1.62)		1.15 (0.47-2.79)	
3^rd ^tertile (least equal)	1		1	

**Self-perceived equality, n(%)**				
Completely equal	156 (84.8)	28 (15.2)	56 (82.4)	12 (17.7)
Relatively equal	189 (82.2)	41 (17.8)	109 (76.2)	34 (23.8)
Not equal	9 (50.0)	9 (50.0)	23 (74.2)	8 (25.8)
**Unadjusted OR (95% CI)**				
Completely equal	5.57 (2.03-15.26)		1.62 (0.59-4.49)	
Relatively equal	4.61 (1.72-12.33)		1.12 (0.46-2.72)	
Not equal	1		1	
Adjusted OR (95% CI)*				
Completely equal	5.19 (1.81-14.82)		1.62 (0.56-4.72)	
Relatively equal	3.96 (1.42-11.05)		1.04 (0.41-2.63)	
Not equal	1		1	

*Note: Adjusted for age, education, occupational grade, and income of the respondents.

There was no statistically significant association between SRH and the gender equality index. However, the gradient for women was in line with our hypothesis, i.e. the odds for good health increased with gender equality, OR 1.54 (CI = 0.6-3.95), for women in the most equal tertile compared to those in the least equal tertile.

The pattern for women remained when we analysed self-perceived equality and SRH, OR 1.62 (95%CI = 0.56-4.72), for women who perceived their relationship to be completely equal compared to women who perceived their relationship as not equal. Further, men who perceived their relationship as completely equal had significantly higher odds of reporting their health as good compared to men who perceived their relationship to be not equal (OR = 5.19 95% CI = 1.81-14.82).

## Discussion

There was no significant association between the self-reported gender equality index and self-rated health, even though women who belonged to the most equal tertile had higher odds of reporting good health compared to those who belonged to the least equal tertile.

For men, there was a statistically significant association between their perception of gender equality in their partner relationship and rating their health as good. For women, the associations were not statistically significant but showed the same directions as men. It is possible that perceiving good health and perceiving equality in your relationship are two sides of the same coin. The question "How do you rate the gender equality in your relationship?" might be understood in a different way than the way that we meant it to be understood. If the question is understood as "Do you have a good relationship?" then it is easy to understand why it is closely linked to self-rated health. Men and women might interpret this question in different ways.

### Inconsistency between the two measures

The differences between self-perceived and self-reported gender equality show different patterns for men and women. Men perceived higher gender equality than they reported, whereas for women we observed the reverse - women perceived lower gender equality than they reported. Men seemed to regard themselves as gender equal in spite of tangible, measureable indicators showing the contrary. It might be difficult for men to acknowledge these differences, as they constitute the beneficiary group. This conclusion could be supported by the fact that women perceive less gender equality even when they report a relatively high consistency of measurable indicators in their daily practice. These two findings highlight the risk of failing to capture the core of the concept of gender equality. Power dynamics are known to be central in relationships [6,37]. Presumably, the distribution of power is central for how people perceive gender equality, and power is difficult to describe and above all difficult to measure.

There might even be a larger difference between the two measurements, as we have been very generous in judging the division of households as equal. If more than 50% of the items were rated as shared equally, we recorded the couple's relationship as equal. Accepting 49% inequality and still ranking the relationship as equal must be considered very indulgent.

This study shows clearly that none of the applied measures is ideal. They tell different stories but are part of the same reality. Men obviously overrate self-perceived gender equality, suggesting that a single question on gender equality does not generate a valid result. On the other hand, the index used overrates equality among women, most likely because important aspects are lacking. A possible solution to this problem might be a combination of measurements - a genuinely gender-equal partnership needs to meet two criteria: (i) it should satisfy reasonable external requirements and (ii) it should be assessed by both partners as equal.

This inconsistency in how gender equality is understood and how it is expressed is observed not only when comparing men and women at an individual level, but also when comparing perceptions of gender equality on structural levels. For instance, although there is a political consensus in Sweden that gender equality is beneficial, there are differences between the parties in its definition [38]. When a societal norm becomes sufficiently strong, it is very difficult to question it publicly, even for political parties. And in the family sphere, societal norms steer decisions even relating to matters that are individual and private.

The fact that 80% of parental leave is taken by mothers is often explained as the most rational choice for the family financially. In a recent study, however, differences in a couple's market productivity did not affect the time spent on household work. This study of market productivity used a theoretical model that assumed that rational economic behaviour explains the division of paid and unpaid work in a family; however, this could not be demonstrated [39].

The strong societal consensus on the goals for gender equality might lead to a duality **- **men and women both want to be progressive and gender equal, at the same time as they are acting rationally and solving domestic problems in the most practical way, which often means conforming to traditional family divisions, with household chores remaining the responsibility of the woman [40,41]. This could possibly also be part of the explanation for the differences found in the current study between the two measures of gender equality. Individuals are often assumed to act in accordance with their self-interests, i.e. what is best for them. Miller suggests that there might be a need for more systematic and direct examination of variance in rational self-interest [42]. The extent to which an individual's actions and/or attitudes reflect a deliberate consideration can vary and this must be taken into account [43].

Further research on variation in self-interest is necessary. The interests that control people's preferences are not always obvious. There might also be differences within groups that are greater than those between groups: even though evaluation of gender equality requires comparison between men and women, we must be aware of possible differences within each group.

The differences between people's own perceptions of gender equality and the self-reported index, attempting to visualize the national policy, can be interpreted in at least two ways. The first interpretation is that people are sufficiently competent to judge themselves whether or not they are gender equal. This view is supported by the idea of the free will and the individual's right to choose. The free will is, however, a complex matter and philosophers have long discussed the liberal paradox formulated by Sen [44]. However, in a couple there is the problem of gender and its traditions and practice [45] . As gender is a matter of relations between people and as women and men are socialized to lead somewhat different lives, it is difficult to speak of free choice: it must always be a question of interpreting different meanings and of having different perspectives. The second interpretation is that this is a good example of couples' ways of "doing gender" in a context where gender equality has high status [46]. Regarding an unequal division of household work as gender equal could be a way of maintaining the prevailing systems - i.e. it could be a way of allowing two different norm systems to exist side by side - on one side, the discourse on gender equality as a goal of Swedish society, and on the other side the discourse of the traditional family norm system with different obligations for men and women. Many families with small children will recognize the situation where all available energy is needed to make everyday life function as smoothly as possible [47]. By describing this situation as self-elected and preferable, the situation is justified, not least to oneself. Moller-Okin argued that the family is not, and cannot be viewed as, the "non-political" area. Social justice is a political goal in a democratic society, and theories of justice need to apply their standards even to the family. Moller-Okin also showed that when the family is argued as belonging to the private sphere, almost all justice theorists assume that "individual" in a family means the male head [15].

### Gender equality and self-rated health

From the previous discussion, we can conclude that self-perceived gender equality among men is not a trustworthy measure. Men who perceive they are gender equal have high odds of reporting good health, but there are no reasons to believe there is a causal relationship. On the contrary, the theory of convergence, even though rather vague, would suggest that gender-equal men could expect to develop some of the ill health typical for women. When applying the index there was no change between the tertiles, and we interpret this to be a more plausible result.

Health is also a concept with many varied explanations. Women in Sweden and many other countries are less healthy than men, almost regardless of the measurement used. They have more days of sickness absence, use more resources of the health care system and report less good health. But at the same time women live longer. So the fact that women acknowledge their ill health may be beneficial for their survival. This is why we have introduced the theory of convergence, suggesting that greater gender equality will benefit both men and women. Women will live healthier lives and men will live longer.

Although Månsdotter acknowledges the potential biological, social and behavioural grounds for the different health patterns of men and women, she also makes clear that the division of male and female affairs is not static. Finding a new pathway to gender convergence might also lead to health convergence for men and women [48]. This development can already be seen in men who participate in their children's upbringing [3]. The long-standing life expectancy gap between men and women appears to be closing in many societies. Men take better care of their health than previously and lead less risky lives, while women adopt more traditionally masculine behaviours such as smoking and alcohol consumption.

For women in the study, health improved with increased gender equality, using both self-reported and self-assessed gender equality; however, the associations were not significant. This pattern was in line with our expectations as we measured self-reported health rather than mortality, and a larger sample might have resulted in significant differences.

### Methodological considerations

In this study, respondents answered the questions individually and on behalf of their partner rather than as a couple. Putting questions to both partners in a relationship is possibly more valid, but very difficult.

The measurement of gender equality in the relationship based on the questions asked in the questionnaire, here called self-reported gender equality, is used as the normative dimension in this study. The responses to the survey questions comprise the respondent's information on how the couple divides responsibilities and time in their family. From this self-reported data we have produced an estimation of gender equality practice in the household, used as a normative comparison to the self-perceived gender equality question. As in our study to be denoted "Gender equal" it was sufficient to answer "We share equally" in 51% of the 14 questions, the risk of overestimating the numbers of gender-equal relationships is obvious. Nevertheless, the number of couples reporting their relationship as gender equal is low compared to how they perceive gender equality in the same relationship. With higher standards for what constitutes an equal relationship, we would have seen even bigger differences.

Although we have used survey data and compared gender equality in quantitative terms, we have been able to show the ambiguity of respondents in relation to this issue. A qualitative research approach would surely enrich the picture and, we believe, capture gender equality in relationships in more diverse ways. Ambiguous as the phenomenon of gender equality seems to be, elaboration on the basis of in-depth interviews would be of great importance. This would enable a deeper understanding of respondents' ideas and perceptions of gender equality in their lives. For method development, the more costly qualitative approach could prove essential; however, the quantitative methods may prove useful when developed with greater sensitivity for ambiguity. In this way, it would be possible to create a cost-effective and easily repeatable quantitative measurement. Our expressed aim to evaluate the gender equality discourse on the basis of both reported practice and self-perceived gender equality involves judging some consequences to be more important than others, and we emphasize that such judgement can and must be challenged. If the way people divide their time, money and responsibility in a relationship is not a sign of gender equality, what is? Have we missed any important issues in the private sphere that add to the issue or are even more descriptive of gender equality?

The use of principal component analysis in deriving the index of equality does not provide any absolute categories of observed inequality, meaning that it is not possible to ascertain the true level of domestic inequality. The index derived by PCA provides a relative comparison between different groups of respondents in terms of other variables, such as health outcome. For example, it would be possible to use the index derived here to study whether health outcome differentiates between households with the least and the greatest equality.

We compared the background characteristics of the included respondents with those who were excluded, i.e. those with complete and incomplete information respectively, and found that respondents with incomplete information tended to be younger, have a lower level of education, and lower income. This might be a source of selection bias in our study.

## Conclusions

In this study, men and women perceived and reported gender equality differently. This makes it necessary to be conscious of the measures used to quantify men's and women's opinions. Both of the measures used have shortcomings. The index for self-reported gender equality likely lacks some dimensions, implying that women are judged by our index to be more equal than they perceive themselves. Furthermore, men perceived themselves to be much more equal than judged by the index. In future studies we thus intend to combine the two measurements. There was no significant association between self-reported gender equality and self-rated health, even though women who belonged to the most equal tertile had higher odds of reporting good health compared to those belonging to the least equal tertile.

For men, there was a significant association between their perception of gender equality and rating their health as good. For women, the association was not significant but showed the same directions.

Gender equality is a matter of equity involving all human beings; the question is therefore too important for society as a whole to be treated as everyone's private matter. We might not yet have found the ultimate measurement; however, we have shown that the single question *"Do you consider your relationship to be gender equal?" *does not meet the standard. We argue here that the self-reported index is a better measurement, particularly when combined with some kind of personal statement on how gender equality is perceived.

## Competing interests

The authors declare that they have no competing interests.

## Authors' contributions

AS carried out the data collection, participated in the analysis, conceived and drafted the manuscript. NN was mainly responsible for the PCA calculations and participated in the draft of the methods and result sections. AÖ and LL participated in all stages of the research process, i.e. in the design of the study, analysis of the data and drafting the manuscript. All authors read and approved of the final manuscript.
